# Organic acids counteract the determinate root development induced by phosphate deprivation in *Arabidopsis thaliana*

**DOI:** 10.1080/15592324.2026.2662710

**Published:** 2026-04-23

**Authors:** Iván Corona-Sánchez, José López-Bucio, José Manuel Villalobos-Escobedo, Saúl Vázquez-Fuentes, Jesús Campos-García, Homero Reyes de la Cruz, Jesús Salvador López-Bucio

**Affiliations:** a Instituto de Investigaciones Químico Biológicas, Universidad Michoacana de San Nicolás de Hidalgo, Morelia, Michoacán, México; b Instituto Tecnológico de Monterrey, Institute for Obesity Research, Monterrey, Nuevo León, México; c Investigador por México SECIHTI-UMSNH, Instituto de Investigaciones Químico Biológicas, Universidad Michoacana de San Nicolás de Hidalgo, Morelia, Michoacán, México

**Keywords:** Plant development, root architecture, phosphate deficiency, iron homeostasis, organic acids

## Abstract

Phosphate (Pi) deficiency impairs plant development and decreases productivity owing to its critical functions in terms of cell structure, metabolism, and photosynthesis. Here, we assessed the determinate growth program of *Arabidopsis* primary roots exposed to Pi scarcity to clarify the role of citrate and malate in the expression of the mitotic cyclin *CycB1* and changes in the meristem, transition zone, and elongation zone. The transfer of seedlings from high-Pi medium to low-Pi medium, but not to high-Pi medium, inhibits root growth, cell division, and elongation, aspects that are restored upon the application of each organic acid. On the other hand, the exhaustion of the meristem under low Pi related to the loss of auxin and the cytokinin response in columella cells is reversed by organic acids, according to the expression of the *DR5:GUS* and *ARR5:GUS* gene constructs, respectively. Histological detection of iron levels at the tip of the primary root and the formation of abundant root hairs, which indicate the repression of mitosis and the advancement of differentiation, decreases with malate and citrate and with the application of ferrozine, an iron chelator, suggesting a relevant role of chelators at the rhizosphere to reduce the adaptive pressure of phosphate scarcity on root growth.

## Introduction

Sustained plant growth and biomass production require proper availability of soil nutrients, which are both perceived and absorbed by root cells. Either nutrient deficiency or excess can lead to physiological adjustments that affect root and shoot growth, defense systems, and tolerance to environmental stress.^1-3^

Phosphorus is a macronutrient readily available as inorganic phosphate (Pi). It is part of essential biomolecules, such as nucleic acids, phospholipids, and nucleoside triphosphates, and takes part in protein phosphorylation-dependent signaling and metabolic regulation.^4^ However, Pi availability in soils is generally low due to the formation of insoluble complexes with metal ions, such as Ca, Fe, and Al, thereby restricting its uptake by roots.^5^ Pi scarcity interferes with mitosis and cell elongation, and thus the growth of the primary root ceases and enters a determinate developmental program that correlates with an enhanced auxin sensitivity.^6^^,^^7^ As a consequence, more lateral roots with denser and longer root hairs are formed, which express Pi transporters and secrete organic acids and phosphatase as an adaptive response to improve Pi acquisition.^6^^,^^8-10^

Considerable effort has been devoted to understand the molecular and cellular mechanisms underlying plant responses to Pi deficiency, where membrane remodeling, endoplasmic reticulum stress, proteostasis, and autophagy are balanced by sumoylation and signaling mediated by MPK3 and MPK6 kinases.^11-13^ Primary root growth inhibition and meristem exhaustion caused by alterations in Pi homeostasis unveiled a critical role of Fe overaccumulation for reactive oxygen species (ROS) production, callose deposition, and impaired cell-to-cell communication.^14-18^

Root exudation of organic acids is an important strategy to cope with nutritional stress. Under Pi limitation, roots secrete malate and citrate, which contribute to Pi mobilization from poorly soluble sources.^8^^,^^19-21^ Citrate and malate show strong affinity for cations, and increase metal tolerance.^22-24^ In spite of the protective roles of malate to heavy metals, its exudation upon low Pi perception at the root tip has been proposed to enhance Fe availability, leading to its uptake, accumulation, and toxicity in the root tip.^15^^,^^16^^,^^18^ This assumption is mainly based on the phenotype of *Arabidopsis* mutants defective on the malate transporter aluminum-activated malate transporter 1 (ALMT1) and its transcriptional activator sensitive to proton rhizotoxicity 1 (STOP1), both of which maintain meristem activity under Pi deprivation, and this response has been linked to reduced malate-dependent Fe accumulation in the root meristem.^18^^,^^25^

Root secretion of malate has been correlated with accumulation of malate-Fe^3+^ complex in the apoplast of root cells, where interconversion of Fe^3+^ to Fe^2+^ catalyzed by the ferroxidases LPR1/LPR2 contributes to the oxidative burst, callose accumulation, and meristem exhaustion.^15^ Blue light also accounts for the interconversion of the ferric and ferrous forms into the apoplast and in accordance, the primary root stoppage of plants experiencing Pi limitation is not inhibited under darkness. Moreover, pH, the amount of Fe, and H_2_O_2_ account for the ability of the photo–Fenton reaction to produce ROS.^26-28^

The citrate transporter ferric reductase defective 3 (FRD3) contributes to Fe efflux and its long-distance transport within plant tissues. Consequently, *frd3* mutants are hypersensitive to Pi limitation and overaccumulate Fe in the root tip, leading to earlier root meristem differentiation.^14^^,^^29-31^ Fe transport from roots to the shoot depends on Fe^3+^-citrate complexes that are uploaded into the xylem, facilitating its mobilization.^23^

Intriguingly, supplements of malate and citrate have been reported to exert variable effects on the primary root under Pi deficiency. Malate supplementation promoted primary root inhibition by increasing Fe accumulation in the root meristem,^18^ whereas citrate transiently sustains primary root growth under Pi scarcity.^31^ These uneven responses are neither logical nor expected given that both compounds can chelate metals and that their exudation to the rhizosphere can protect roots from metal toxicity.^18^^,^^21^^,^^31-33^

Since malate and citrate often show comparable roles in plant adaptation to nutrient deficiency, the apparent mismatch in their effects on primary root responses to Pi limitation remains unresolved. In this work, we analyzed the previously reported determinate root growth induced by Pi scarcity in *Arabidopsis* to assess the role of malate and citrate in meristem dynamics, root zonation, hormonal responses, and its relationship with Fe toxicity. We found a comparable effect of both organic acids in conferring resistance to low Pi-driven determinate root growth, which underlies the importance of these metal chelators in the rhizosphere for adaptation to phosphate scarcity and promoting metal tolerance.

## Materials and methods

### Plant material and growth conditions

*Arabidopsis thaliana* ecotype Columbia-0 (Col-0), reporter lines *CYCB1;1:uidA*,^34^ *DR5:GUS*,^35^ and *ARR5:GUS*^36^ were used to monitor mitotic activity, auxin response, and cytokinin response, respectively. Seeds were disinfected for 5 min in 70% (v/v) ethanol followed by 7 min in 20% (v/v) commercial bleach and rinsed five times with autoclaved distilled water. After stratification at 4 °C for 2 d in darkness, seeds were sown on 0.2× Murashige and Skoog (MS) medium supplemented with 0.6% (w/v) sucrose and 1% (w/v) agar. Plates were positioned vertically and seedlings were grown at 22 °C under long-day conditions (16 h light/8 h darkness photoperiod; 100–120 µmol m^−2^ s^−1^). Five-day-old seedlings were transferred to fresh media containing the indicated treatments and grown for an additional 5 d period.

### Phosphate, iron, and organic acid treatments

Phosphate-sufficiency conditions refer to media supplemented with 250 µM potassium phosphate (Pi), whereas phosphate-deficient conditions refer to 1 µM Pi application. The media were supplemented with malate or citrate at final concentrations of 0, 40, 80, or 160 µM, as specified for each experiment. For the iron treatments, the seedlings were grown either under standard iron supply (Fe-EDTA) or under iron-limiting conditions generated by omitting iron from the medium and/or supplementing it with 200 µM ferrozine to chelate residual Fe. All media were adjusted to pH 5.7 prior to autoclaving.

### Root growth and morphometric analyses

Primary root length was measured after 5 d of treatment on vertically grown seedlings using a ruler. All other morphometric parameters, including meristem length, transition zone length, elongation zone length, and cell length in the differentiation zone, were determined from calibrated digital micrographs. Measurements were performed using ImageJ software. For each experiment, at least five independent biological replicates were analyzed (*n* ≥ 5).

### Histochemical GUS reaction and microscopy

For histochemical GUS detection, the seedlings were incubated for enzymatic reaction into a solution containing 5-bromo-4-chloro-3-indolyl-*β*-D-glucuronic acid (X-Gluc), 100 mM sodium phosphate buffer (pH 7.0), 0.5 mM potassium ferricyanide, 0.5 mM potassium ferrocyanide, and 0.1% (v/v) Triton X-100 at 37 °C until the appearance of a blue color indicative of substrate hydrolysis. The samples were cleared through an ethanol series prior to imaging. Differential interference contrast (DIC) images were acquired using a Leica DM500B microscope. Imaging parameters were maintained constant within each experiment.

### Perls/DAB iron detection

Iron localization was assessed using Perls staining followed by diaminobenzidine (DAB) intensification to increase Fe³⁺ detection. Briefly, seedlings were vacuum-infiltrated in freshly prepared Perls solution (equal volumes of 4% [w/v] potassium ferrocyanide and 4% [v/v] HCl) and incubated for 30 min at room temperature. Samples were rinsed thoroughly with distilled water.

For signal intensification, seedlings were incubated in DAB solution (0.025% [w/v] diaminobenzidine tetrahydrochloride in 0.1 M sodium phosphate buffer, pH 7.0, supplemented with 0.005% [v/v] H₂O₂) for 10–15 min. The reaction was stopped by rinsing in distilled water. Stained seedlings were imaged using differential interference contrast (DIC) microscopy, and identical acquisition settings were maintained across treatments.

### Free iron quantification

Iron quantification was performed by a colorimetric method as previously reported.^37^ Briefly, a linear calibration curve was constructed using liquid MS 0.2× with increasing concentrations of FeSO_4_ (0, 5, 10, 15, 20, 25, and 30 µM) with a final pH media of 5.7 and 4.9 mM of ferrozine. MS 0.2× aliquots with Pi sufficiency (250 µM) or deficiency (1 µM) with or without citrate and malate (160 µM) were mixed with 400 µL of 4.9 mM of ferrozine and the absorbance was measured at 562 nm. Fe concentration was determined using the initial calibration curve.

### Statistical analysis

Data are presented as mean ± standard error (SE). Statistical analyses were performed using one-way ANOVA followed by Tukey's post hoc test to determine significant differences among treatments. Statistical significance was defined as *p* < 0.05. Normality and homogeneity of variance were verified prior to ANOVA analysis. All the statistical analyses were conducted using GraphPad Prism (GraphPad Software, San Diego, CA, USA).

## Results

### Malate and citrate rescue primary root growth under Pi deficiency in *Arabidopsis*

**Figure 1.**
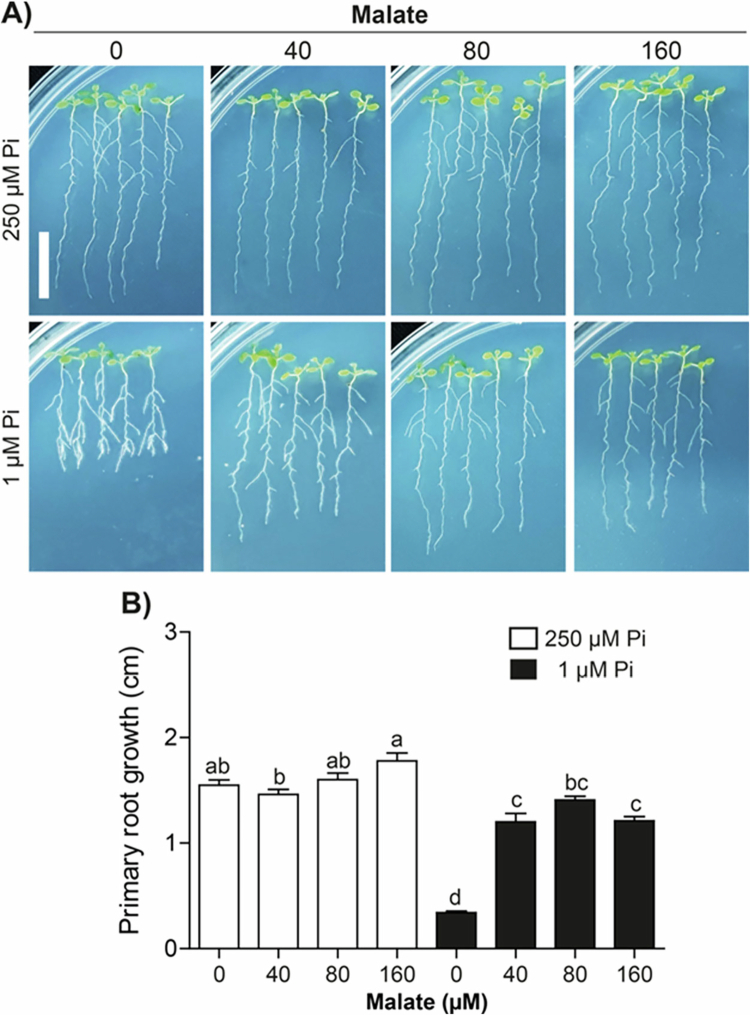
Malate normalizes primary root growth of *Arabidopsis* plants under phosphate deficiency. (A) Representative images of plants under Pi sufficiency (250 µM) or deficiency (1 µM) without (0 µM) or supplemented with 40, 80, and 160 µM malate. Scale bar = 1 cm. (B) Quantification of primary root growth attained after the transfer of seedlings to contrasting Pi conditions and treated with malate, bars represent the mean ± standard error (SE). *n* = 30 plants from two independent experiments. Different letters indicate statistically significant differences among treatments as determined by one-way ANOVA followed by Tukey's post hoc test (*p* < 0.05).

Root-secreted malate has been implicated in Fe uptake and accumulation in the root meristem under low Pi conditions.^18^ To assess the precise role of this organic acid for the determinate developmental program elicited by Pi scarcity, *Arabidopsis* seedlings were grown under Pi sufficiency for 5 d and then transferred to media with 250 µM Pi (Pi sufficiency) or 1 µM (Pi limitation), supplemented with increasing concentrations (0, 40, 80, and 160 μM) of malate. In plants transferred to high-Pi medium, the primary roots showed rapid and sustained growth that was not significantly altered by the enrichment of the medium with malate. In contrast, transferring the plants to low-Pi medium caused repression of root growth and a marked change in root system architecture, including the formation of lateral roots near the apex (Figure 1a). In this case, the application of malate from 40 to 160 µM reactivated root growth (Figure 1b), and no visible lateral roots appeared near the root tip (Figure 1a, b).

**Figure 2.**
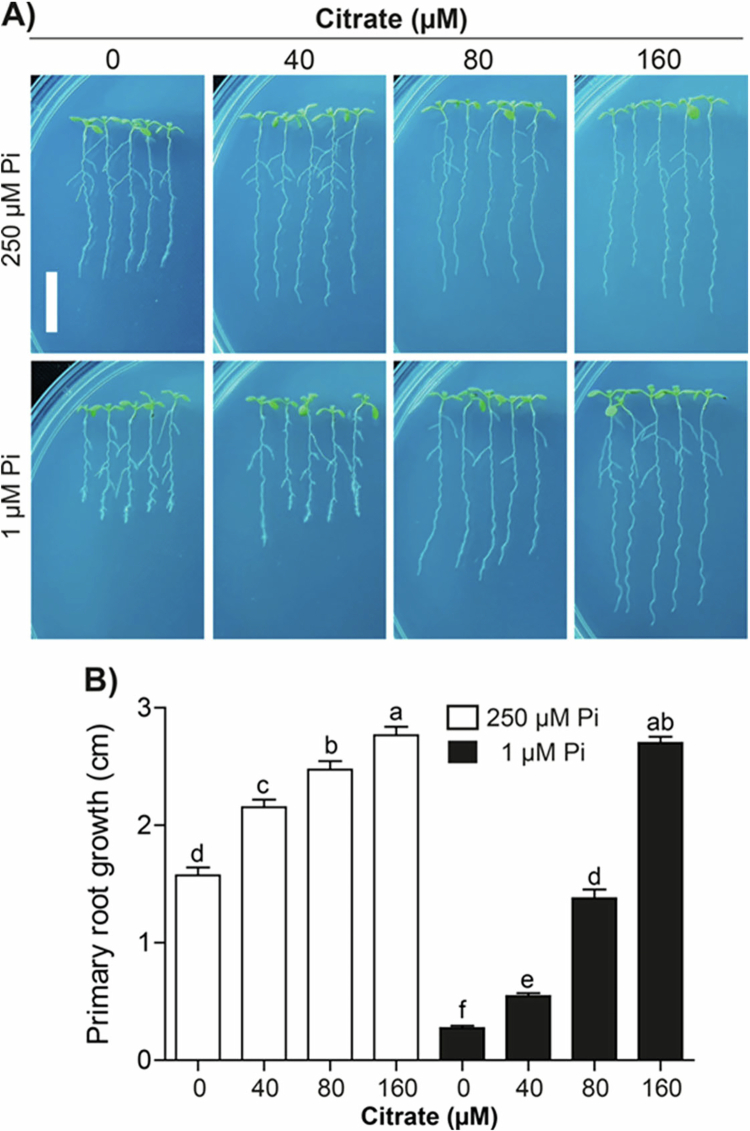
Citrate promotes primary root growth of *Arabidopsis* plants under high-Pi and low-Pi conditions. (A) Representative images of plants under Pi sufficiency (250 µM) or deficiency (1 µM) without (0 µM) or supplemented with 40, 80, and 160 µM citrate. Scale bar = 1 cm. (B) Quantification of primary root after transfer to contrasting Pi media and citrate treatments; bars represent the mean ± standard error (SE). *n* = 30 plants from two independent experiments. Different letters indicate statistically significant differences among treatments as determined by one-way ANOVA followed by Tukey's post hoc test (*p* < 0.05).

Malate has two carboxyl groups in its structure, whereas citrate includes three carboxyl groups, which are key for their activity as chelators. Next, the effect of citrate application (0, 40, 80, or 160 μM) was analyzed in plants transferred to media with high or low Pi. Noteworthy, citrate treatment enhanced the growth of the primary root in plants in high-phosphate medium in a dose-dependent manner, an effect that was not observed with malate, and reactivated the growth of the primary root in plants under Pi limitation, reaching further the length observed in plants in high-Pi and high-citrate supplements (Figure 2a, b). These results indicate that citrate acts as a root growth promoter in these experiments regardless of the Pi concentration in the medium.

### Malate and citrate support root meristem activity and cell elongation under Pi scarcity

**Figure 3.**
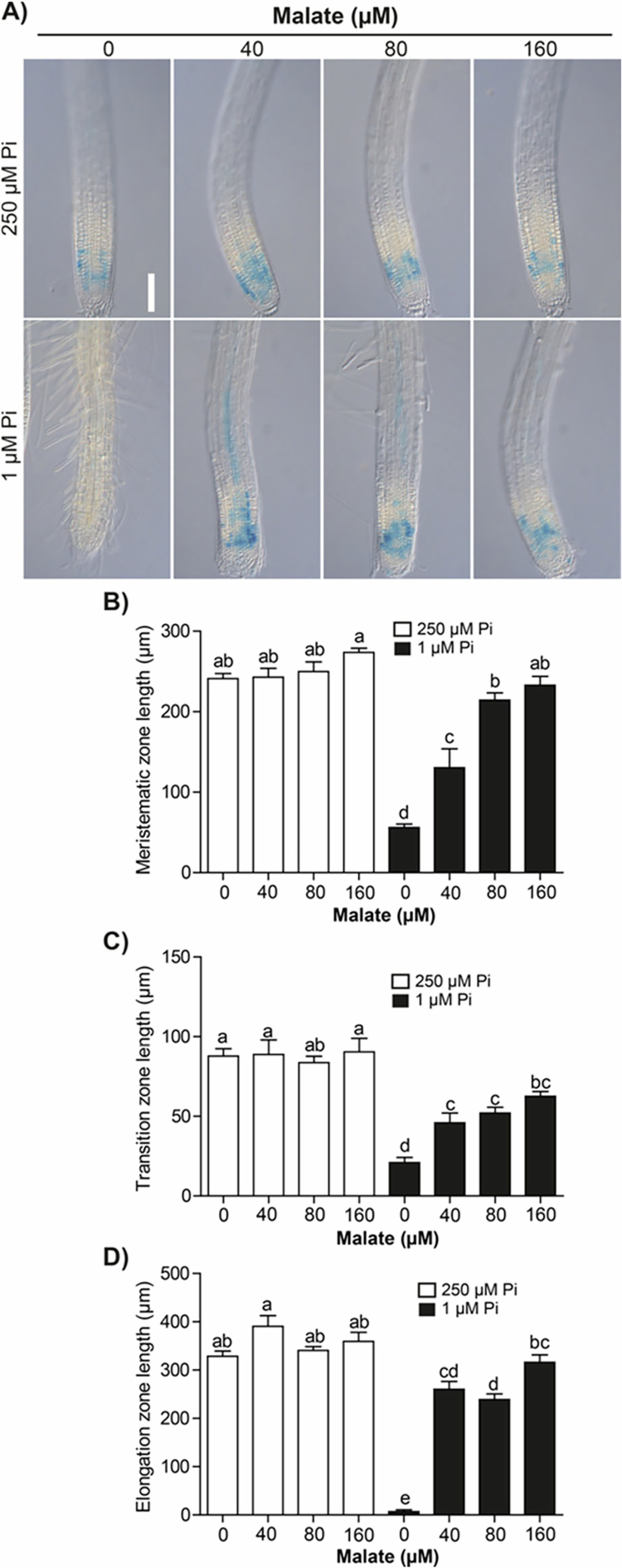
Malate maintains the proliferative activity and root tip organization of plants under phosphate deficiency. (A) Expression of *CycB1;1:uidA* under Pi sufficiency (250 µM) or deficiency (1 µM) without (0 µM) or supplemented with 40, 80, and 160 µM malate. Scale bar = 100 µm. Length of (B) meristematic, (C) transition, and (D) elongation zones from the primary root tip in the indicated treatments. Bars represent the mean ±  standard error (SE). *n* = 15 plants from two independent experiments. Different letters indicate statistically significant differences among treatments determined by one-way ANOVA followed by Tukey's post hoc test (*p* < 0.05).

**Figure 4.**
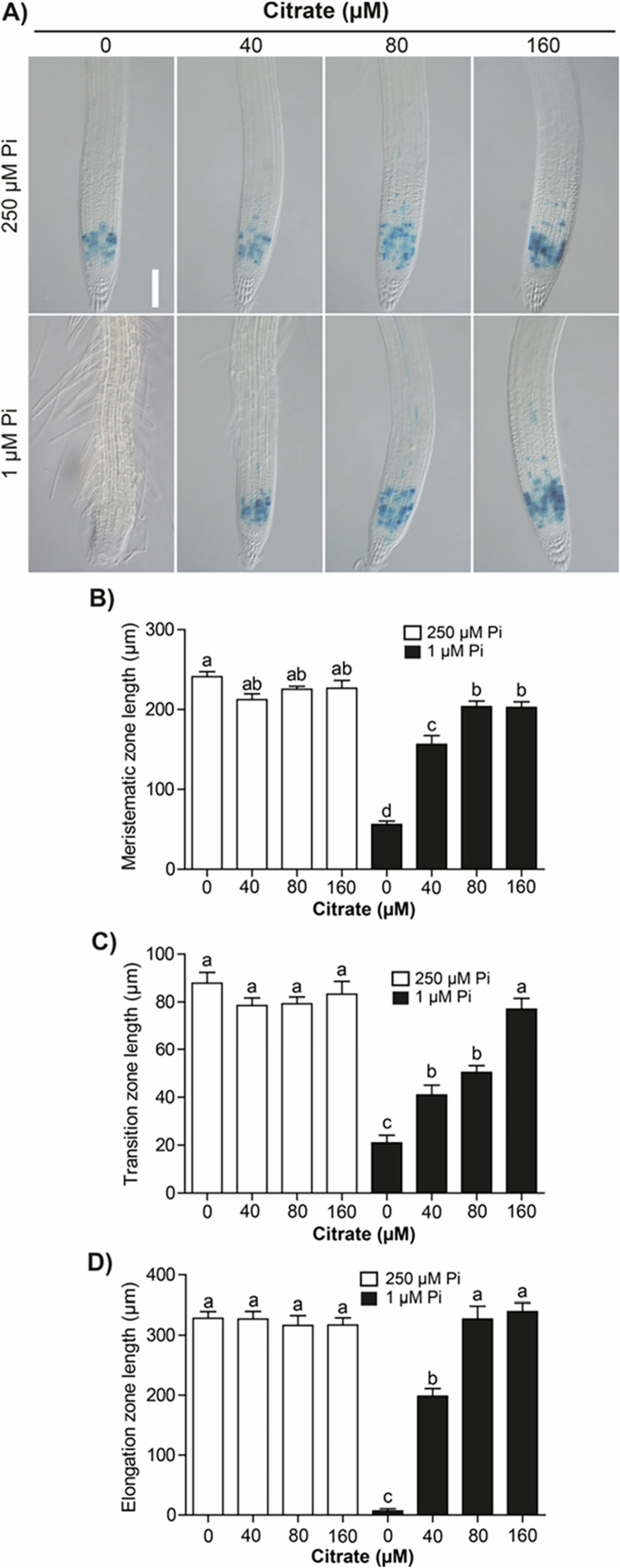
Citrate supports the cell proliferative activity and root tip structure of plants under phosphate deficiency. (A) Expression of *CycB1;1:uidA* under Pi sufficiency (250 µM) or deficiency (1 µM) without (0 µM) or supplemented with 40, 80, and 160 µM citrate. Scale bar = 100 µm. Length of (B) meristematic, (C) transition, and (D) elongation zones from the primary root tip in the indicated treatments. Bars represent the mean ± standard error (SE). *n* = 15 plants from two independent experiments. Different letters indicate statistically significant differences among treatments determined by one-way ANOVA followed by Tukey's post hoc test (*p* < 0.05).

Pi scarcity affects cell division and elongation, leading to root growth stoppage. The role of organic acids in supporting root growth under contrasting Pi supply was analyzed by evaluating the expression of *CycB1;1:uidA,* a marker of cell proliferation in the meristem, and by measuring the length of the meristem and the transition and elongation zones. Under Pi sufficiency conditions, *CycB1* expression does not change upon malate supplementation; however, under Pi deficiency, where *CycB1* expression in the meristem is completely suppressed, malate supplementation restored the mitotic activity to levels comparable to those observed under Pi sufficiency conditions (Figure 3a). Citrate supplementation also restored *CycB1* expression in seedlings grown under Pi limitation. Noteworthy, 160 µM citrate under either Pi sufficiency or deficiency conditions strongly induced the expression of the mitotic cyclin, which correlated with primary root growth (Figure 4a).

The length of the meristematic (Figure 3b and, 4b), transition (Figure 3c and 4c), and elongation zones (Figure 3d and 4d) did not change by malate or citrate supplementation under Pi sufficiency, but the reduction in the length of such zones imposed by Pi limitation was reverted by malate or citrate, respectively (Figure 3b–d and 4b–d). These data unveil for the first time the influence exerted by Pi availability on root zonation and the protective effects of organic acids to support cell division and elongation under low-Pi stress.

### Malate and citrate sustain auxin and cytokinin response in columella cells under low Pi conditions

In plants experiencing low-Pi stress, entry into determinate root growth correlated with the loss of the auxin response in columella cells.^6^ To assess whether the hormonal dynamics at the root tip may be normalized upon treatment of low-Pi-grown seedlings with organic acids, *DR5:GUS* and *ARR5:GUS* were used to monitor the local auxin and cytokinin response, respectively.

**Figure 5.**
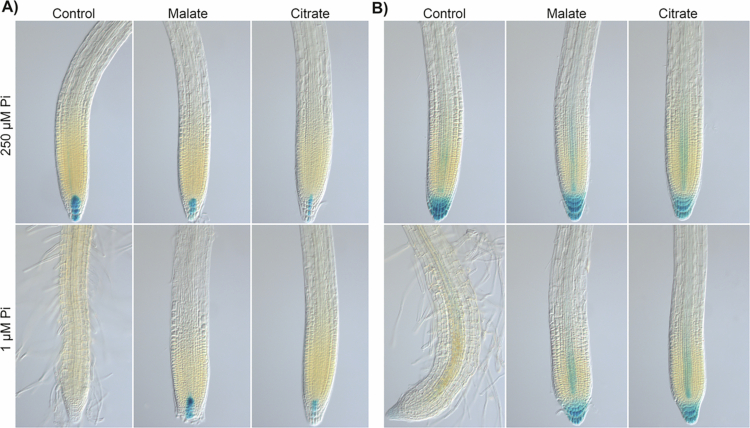
Malate and citrate restore auxin and cytokinin signaling in the primary root of *Arabidopsis* plants under phosphate deficiency. Representative images from primary roots of reporter lines (A) *DR5:uidA* and (B) *ARR5:uidA* for auxin and cytokinin signaling, respectively, under Pi sufficiency (250 µM) or deficiency (1 µM) without (0 µM) or supplemented with 160 µM of malate and citrate. Images are representative of 12 seedlings analyzed. The experiment was repeated twice with comparable results.

In plants transferred to high-Pi medium, the expression of *DR5:GUS* and *ARR5:GUS* was specifically localized in columella cells, as evidenced by the blue color in this area (Figure 5a, b). Treatment with 160 µM malate or citrate shows the expression of the markers. On the other hand, in both genotypes, transfer to low Pi caused the loss of the meristematic and elongation zones, and instead, differentiation was promoted as evidenced by the formation of root hairs at the root tip (Figure 5a, b). Treatment with malate or citrate reverses the differentiation, and now the expression of *DR5:GUS* and *ARR5:GUS* is observed in the columella (Figure 5a, b). These results show the establishment of hormonal activity by organic acids in plants subjected to phosphate deficiency.

### Iron accumulation by Pi deficiency in the root tips is reversed by malate and citrate

**Figure 6.**
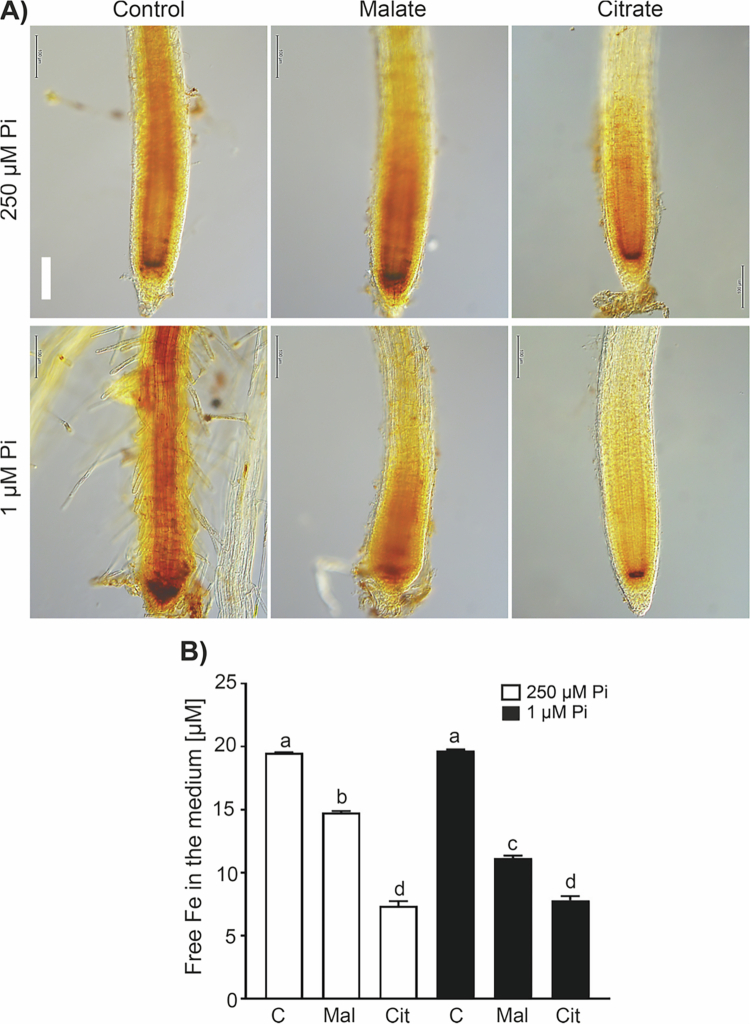
Malate and citrate prevent iron accumulation in the root tip under phosphate deficiency and diminish free Fe in MS 0.2× medium. (A) Representative images of the root tip stained by Perls/DAB from plants under Pi sufficiency (250 µM) or deficiency (1 µM) without (0 µM) or supplemented with 160 µM malate or citrate. Images are representative of 12 seedlings analyzed. Scale bar = 100 µm. The experiment was repeated twice with comparable results. (B) Free iron concentration in liquid MS 0.2× media with contrasting Pi conditions, Pi sufficiency (250 µM) or deficiency (1 µM), and without (0 µM) or supplemented with 160 µM citrate or malate. Bars represent the mean of the Fe concentration ± standard error (SE). *n* = 3. Different letters indicate statistically significant differences among treatments as determined by one-way ANOVA followed by Tukey's post hoc test (*p* < 0.05).

The determinate root development under low-Pi conditions has been attributed to the accumulation of toxic Fe concentrations in root tips.^14^^,^^15^ To assess whether malate and citrate affect Fe accumulation and distribution in root tips in response to Pi availability, we analyzed the iron levels by Perls/DAB histochemical staining. In Pi-sufficiency conditions, both organic acids, but more strongly citrate diminished Fe accumulation in *Arabidopsis* roots tips (Figure 6a). Under Pi limitation, where the primary roots accumulated most of the Fe at the stem cell niche and along the vasculature extending toward the distal tip, malate and citrate supplementation prevented Fe accumulation (Figure 6b). Fe accumulation in the roots correlates with the content of free Fe in the medium, which is diminished in both Pi sufficiency and deficiency conditions by malate and citrate (Figure 6b). These data support the role of organic acids as inhibitors of Fe uptake and/or accumulation in *Arabidopsis* primary roots.

### Fe-induced determinate root growth and meristem differentiation under low-Pi conditions is prevented by the iron chelator ferrozine

**Figure 7.**
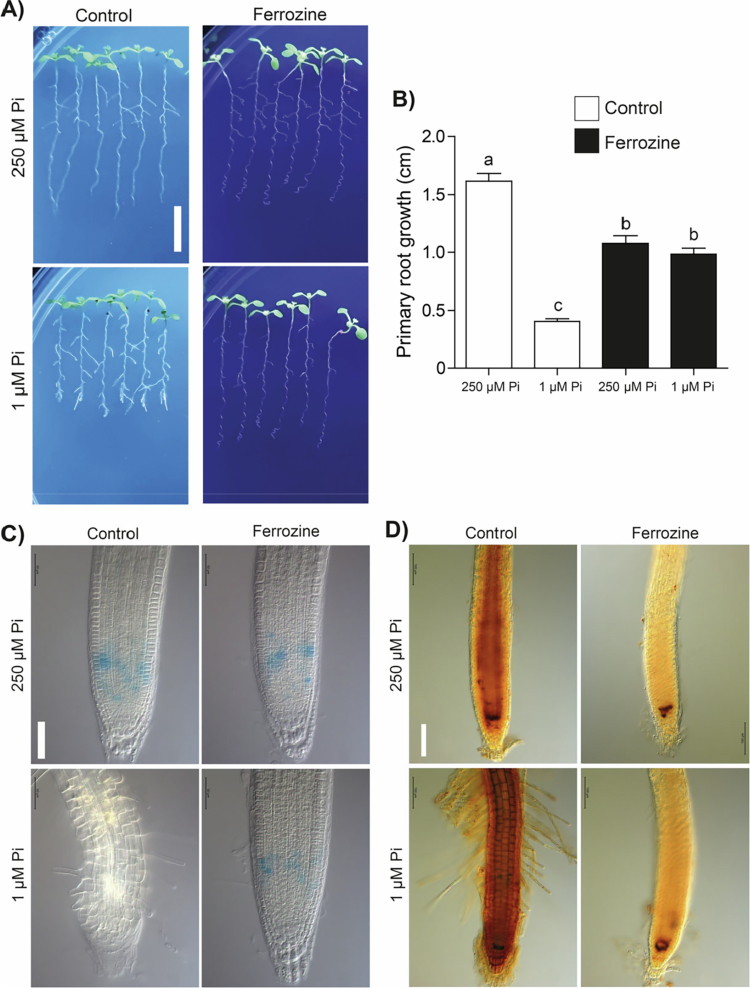
Iron chelation prevents primary root inhibition in plants under phosphate deficiency and decreases iron accumulation in the root tip. (A) Representative images of plants under Pi sufficiency (250 µM) or deficiency (1 µM) without (control) or supplemented with 200 µM ferrozine. Scale bar= 1 cm. (B) Primary root length in plants from the contrasting Pi conditions and treated with ferrozine, bars represent the mean ± standard error (SE). *n* = 30 plants from two independent experiments. Different letters indicate statistically significant differences among treatments as determined by one-way ANOVA followed by Tukey's post hoc test (*p* < 0.05). (C) Representative images from primary roots of proliferation reporter line *CycB1;1:uidA*. Scale bar= 50 µm. And (D) representative images of the root tip stained by Perls/DAB from plants under Pi sufficiency contrasting conditions with or without ferrozine. Scale bar = 100 µm.

Ferrozine (4, 4′-[3-(2-pyridinyl)-1, 2, 4-triazine-5, 6-diyl]bis-benzenesulfonic acid) functions as a high-affinity chelator that selectively binds ferrous iron, forming a stable, intensely colored complex. To assess Fe levels in root cells, 200 µM ferrozine treatment was applied to the high-Pi and low-Pi media, and the root growth, meristem activity, and Fe in roots was analyzed. Applying this iron chelator, the *Arabidopsis* seedlings became resistant to the primary root inhibition caused by Pi deficiency, showing active meristems (Figure 7a–c) and reduced iron accumulation (Figure 7d). Taken together, our data point to a critical role for iron chelation in keeping the meristematic activity and root growth under Pi scarcity.

## Discussion

The root tip senses nutrient levels in the soil and triggers changes in the root system architecture when Pi is scarce.^2^^,^^13^ Local perception of low Pi levels causes physiological and molecular responses aimed at optimizing Pi uptake and utilization. These responses include the repression of root growth, the induction of lateral roots and root hairs, Pi transporter expression and exudation of phosphatase to increase Pi absorption. Research over the last 10 y concluded that the primary root growth stoppage occurs by termination of cell proliferation in the meristem, which results from Fe overaccumulation, and the underlying oxidative burst that disrupts stem cell activity.^14^^,^^15^^,^^38^

Previous studies highlighted the role of organic acids in nutrient availability, uptake, and accumulation in plants. Environmental stress, including nutrient limitation and toxic metals, such as aluminum, triggers organic acid exudation by roots,^39^ which may act as metal chelators at the rhizosphere. Malate and citrate are by far the most investigated organic acids, and owing to their carboxylic groups, they are proposed to act in metal chelation.^40^^,^^41^ It is believed that malate and/or citrate exudation triggered by Pi deficiency helps to mobilize Pi from metal complexes around roots, including Fe–P precipitates, thereby increasing phosphorus availability. However, in a Pi scarcity scenario, malate induces Fe accumulation in apoplastic cells throughout the meristem, where ROS burst by the action of LPR1/LPR2 ferroxidases or depending on the photo–Fenton reaction, which interconverts Fe^3+^ to Fe^2+^ causes callose deposition, meristem differentiation, and primary root growth arrest.^26^^,^^28^ Determinate root growth under low Pi is alleviated not only by changing the experimental light conditions but also by mutation of STOP1 and ALMT1, two critical proteins for malate secretion, and the ferroxidases LPR1 and LPR2. In contrast to WT plants, mutant plants defective on the above-mentioned genes fail to enter the determinate root growth program induced by Pi deficiency. It is important to keep in mind that the resistance observed in *stop1* and *almt1* mutants to low Pi was reversed by 1 mM malate supplementation according to previous reports.^18^^,^^25^

Citrate, which is essential for Fe mobilization from roots to leaves when exuded to the rhizosphere, also promotes Fe uptake in plants experiencing Pi and Fe scarcity.^29^^,^^30^^,^^38^ Therefore, our initial expectation was that organic acids could be involved in Fe dynamics, impairing root growth and exacerbating meristem disruption during Pi scarcity. Certainly, this was not the case, and either malate or citrate could effectively protect *Arabidopsis* roots from growth repression and enter into determinate root development in plants transferred to low-Pi medium.

An analysis of *almt1* and *stop1* mutants by Balzergue et al.^42^ failed to detect differential Fe accumulation in either mutant when compared to WT plants. Interestingly, the authors concluded that the primary root inhibition modulated by STOP1 and ALMT1 depend on mechanisms related to cell wall processes at the expansion zone of the primary root, which is likely uncoupled from the response in the root meristem at early times of low Pi exposure. Mora-Macías et al.^18^ and Wang et al.^25^ showed that the resistance of both mutants to Pi deficiency was normalized by malate supplementation and depends on the Fe accumulation in the apoplast of root tip cells, ROS burst and underlying cellular injury. Although these authors showed comparable responses to Pi limitation, in their analyses of Fe levels in the meristem of *stop1* and *almt1* the reports had some inconsistencies, for instance, levels of Fe assayed by Mora-Macías et al.^18^, which indicated low Fe accumulation in the root meristem of the mutants under either Pi sufficiency or limitation, whereas Wang et al.^25^ did not find differences in Fe accumulation in the mutants under neither Pi sufficiency nor Pi limitation when compared to the WT. These data make necessary to revisit the roles potentially played by organic acids in the rhizosphere and plant tissues regarding Pi and Fe dynamics and distribution.

The concentration of malate supplied by Mora-Macías et al.^18^, which reverted *almt1* and *stop1* responses to low Pi was 1 mM, a very high amount considering physiological concentrations. In our experiments, it was 40 µM malate, a 25-fold lower concentration sufficient to enable root growth of WT plants in low-Pi medium, it could be possible that 1 mM malate causes a toxic effect depending on the growth conditions that explains the root growth repression. Wang et al.^43^ reinforced this notion, since supplementation of 0.5 and 1 mM malate inhibited primary root growth in Pi-deprived seedlings. However, the function of the organic acid strongly depended on the acidic condition of the medium, as shown by the pH being lower than 5.5, in which WT plants and *ALMT1-*overexpressing lines had increased primary root growth inhibition. On the other hand, when malate is supplied to plants under Pi limitation at pH greater than 5.5, WT plants partially resisted the inhibitory effect while *ALMT1-*overexpressing lines were inhibited by malate in concordance with its high acidification capacity, and finally at pH 6.5 the inhibition of the primary root of *ALMT1-*overexpressing lines was as resistant to Pi deficiency as that of WT plants.^43^ These results are in agreement with our findings about the protective effect of malate in the primary root of plants under Pi limitation, which depends on the tested pH conditions. Considering that malic acid has pK values of 3.4 and 5.1, at pH values below 5.1, there is a higher proportion of malate-1 than malate -2, while at pH values above 5.1, the proportion of malate-2 is greater than that of malate-1. Consequently, the interactions between malate-1 and the ferric ion will be weaker, and the structures formed between them will be less complex, allowing some ferric ion availability at pH values below 5.1. Conversely, at pH values above 5.1, the interactions between malate-2 and the ferric ion will be stronger, and the structures formed will be more complex, reducing the availability of the ferric ion, preventing Fe distribution into the root and maintaining the activity of the meristem.

In contrast to malate, the effects of citrate in the low Pi root architecture configuration were less understood. In this regard our data show that it effectively alleviates primary root growth inhibition under Pi deficiency. Citrate function could be related to its deprotonated states at pH 5.7 and above as occurs in malate, but with three possible reduced forms -1, -2, and -3, which effectively chelates Fe in the medium. We propose that malate- and citrate-mediated Fe chelation avoid entrance of toxic Fe levels into the stem cell niche, the root apical meristem, and/or the cell elongation zone, thus contributing to root growth under Pi scarcity. Our data highlight a dual and context-dependent role for organic acids in the crosstalk between phosphate and iron homeostasis.

Recent reports highlighted the importance of move away Fe^3+^ from the root tip to prevent Fenton reactions, which induce ROS and callose accumulation. The ferric reductases CYBDOM help to detoxify Fe^3+^ under Pi limitation. CYBDOM proteins are ascorbate-dependent metalloreductases, which transform Fe^3+^ to Fe^2+^ and helps to mobilize iron from the apoplast, prevents ROS accumulation and protect the primary root from Fe toxicity; thus, plants carrying mutations in genes encoding CYBDOM proteins are hypersensitive to low Pi by increasing Fe and callose at the root tip.^44^^,^^45^ In spite of the mentioned enzymatic activities induced by Pi deficiency, which prevent ROS accumulation in the meristem, Pi limitation also induce the efflux of protons and malate by H(+)-ATPases and ALMT1, respectively, which increase rhizosphere acidification and may release nutrients, including Fe, whose availability triggers Fe-dependent toxicity in the root.^43^^,^^46^ However, under certain physicochemical conditions, such as neutral or alkaline pH, the reduced forms of malate and citrate could bind Fe and prevents its availability and accumulation such as reported by Wang *et al.* 2025.^43^ Therefore, considering our data and previous reports, under our growth conditions, we propose that given the affinity of citrate and malate for binding Fe at pH values above 5.7, their protective effects may result from Fe chelation in the rhizosphere, thereby preventing Fe toxicity. The evidence supporting this idea includes the Fe accumulation patterns observed under Pi deficiency, which are similar to the Fe levels observed in the meristem of plants under Pi deficiency supplemented with ferrozine, an iron-chelating agent. The primary root growth inhibition response to Fe accumulation in the meristem during Pi limitation is consistently bypassed when the growth medium lacks Fe; under these conditions, Fe accumulation is markedly reduced, and cell proliferation and meristem remains active, sustaining primary root growth.
